# Comparative Analysis of Basal vs. Apical Left Ventricular Aneurysms: Impact on Ejection Fraction and Cardiac Function

**DOI:** 10.3390/medicina60101578

**Published:** 2024-09-26

**Authors:** Slobodan Tomić, Stefan Veljković, Armin Šljivo, Tatjana Raičković, Jovana Lakčević, Olivera Đokić, Ana Peruničić, Aleksandra Nikolić, Milovan Bojić

**Affiliations:** 1 Cardiovascular Institute “Dedinje”, 11040 Belgrade, Serbia; bobantomic99@gmail.com (S.T.); tatjana.raickovic@gmail.com (T.R.); jovana.lakcevic@gmail.com (J.L.); oljaisara@gmail.com (O.Đ.); anaperunicic@hotmail.com (A.P.); nikolicdrsasa@gmail.com (A.N.); dedinje@ikvbd.com (M.B.); 2 Faculty of Medicine, University of Banja Luka, 78000 Banja Luka, Bosnia and Herzegovina; 3 Clinical Center of University of Sarajevo, 71000 Sarajevo, Bosnia and Herzegovina; 4 Faculty of Medicine, University of Belgrade, 11000 Belgrade, Serbia

**Keywords:** aneurysm, cardiac volume, cardiac surgery, echocardiography

## Abstract

*Background and Objectives*: Left ventricular aneurysm (LVA) is associated with a decline in cardiac function, evidenced by a lower ejection fraction (EF), due to the reduction in the proportion of functional myocardium. The left ventricular end-diastolic volume (LVEDV), the left ventricular aneurysm volume (LVAV), and the LVAV/LVEDV ratio show a strong correlation with the EF. The aim of this study was to determine LVA characteristics post-myocardial infarction (basal vs. apical) and to evaluate the impact of aneurysm volume in diastole (LVAVd), aneurysm area in diastole (LVAAd), and their respective ratios with LVEDV and area (LVEDA) on the EF, in order to identify the most critical predictive factors for assessing and managing the negative impact of aneurysms on cardiac function. *Materials and Methods*: This observational study included post-infarction LVA patients at the “Dedinje” Cardiovascular Institute in Belgrade, Serbia, undergoing routine transthoracic echocardiography. Echocardiography assessed volumes (LVEDV, LVESV, LVAVd, LVAVs) and areas (LVAAd, LVAAs, LVEDA, LVESA) using the area–length method. The ratios (LVAVd/LVEDV, LVAVs/LVESV, LVAAd/LVEDA, LVAAs/LVESA) were derived from these measures. The left ventricular EF was calculated using Simpson’s method. *Results*: Basal aneurysms showed a significantly smaller LVAVd (*p* = 0.016), LVAAd (*p* = 0.003), and LVAAs (*p* = 0.029) compared to apical aneurysms, indicating that basal aneurysms are smaller in size. However, there was no significant difference in the EF and overall LV volumes between the groups, although the basal aneurysm group had a slightly higher EF and end-diastolic volume, with a slightly lower end-systolic volume. Furthermore, when comparing the correlation between the EF and the LVAVd, the LVEDV, and the LVAVd/LVEDV ratio, the results indicate that the LVAVd had the greatest impact on the EF (−0.695), followed by the LVAVd/LVEDV ratio (−0.637), and the lowest correlation is between the EF and LVEDV. A similar relationship is observed when comparing the EF with the LVESV, the LVAVs, and the LVAVs/LVESV ratio. *Conclusions*: Basal aneurysms are significantly smaller than apical ones, yet EF and LV volumes remain similar between the groups, with the EF being slightly higher in the basal group. In cases of LVA, LVAVd shows the strongest negative correlation with the EF, indicating its significant impact on systolic function, followed by the LVAVd/LVEDV ratio, with the weakest correlation seen between the EF and LVEDV.

## 1. Introduction

The development of a left ventricular aneurysm (LVA) is associated with a decline in cardiac function and a reduced left ventricular ejection fraction (EF) [1,2], as the aneurysm decreases functional myocardium and may expand during systole, impairing ventricular efficiency. LVA typically arises as a complication of myocardial infarction due to left ventricular remodeling, leading to LV enlargement, reduced cardiac output, heart failure, and worsening cardiac function [3,4]. Post-infarction aneurysms predominantly localize to the apical or apical-anterior regions in 59.5% of cases, with apical-septal, apical-inferior, and apical-septal-inferior locations each accounting for 7.1%, and postero-basal locations also comprising 7.1% [5]. A multicenter study corroborates these findings, reporting anterolateral aneurysms in 85.7% of cases and infero-posterior ones in 14.3% [6,7]. Visser et al. [8] found that most aneurysms in 422 myocardial infarction patients were antero-apical. Among 269 patients who underwent aneurysmectomy, over 80% had apical aneurysms, while 5–10% had posterior aneurysms [9]. Similarly, a study of 158 patients over six years reported that 96% had anterior aneurysms and 4% had posterior [10]. Additionally, a study involving 193 patients with akinetic and dyskinetic aneurysms found 90% had anterior aneurysms, 9% had posterior, and 0.5% had lateral aneurysms [11].

The volume of a left ventricular aneurysm (LVAV) can be accurately measured with two-dimensional echocardiography, aiding in evaluating its impact on cardiac function, guiding surgical decisions, and assessing its prognostic value [12,13,14]. However, the effect of LVAV on cardiac function has been infrequently documented [15]. A significant correlation between the left ventricular end-diastolic volume (LVEDV) and EF in the presence of an aneurysm has been emphasized. However, contradictory findings have also been reported [1], suggesting that the formation of LVA induces alterations in the geometry of the LV, thereby diminishing the influence of LVEDV on cardiac function. Hong and colleagues [15], in an experimental study on rabbits by ligating the anterior left descending and circumflex arteries and forming an LVA, established significant correlation relationships between the aneurysm volume and LVEDV in relation to the EF. Specifically, the correlation between the aneurysm volume to left ventricular end-diastolic volume (LVAV/LVEDV) ratio and EF was higher. According to the mechanical theory, the LVAV reflects the extent of myocardial injury. In this sense, the absent (“conflicting”) movements of the aneurysm region reduce the global contractility of the LV. On the other hand, LVAV affects the volume load of the LV, so LVAV can be used as a parameter to assess heart function. This means that the LVAV-to-LVEDV ratio to some extent reflects cardiac output, with a higher ratio indicating reduced cardiac output, due to inefficient LV ejection and a decreased EF from aneurysm expansion during systole. Its superiority lies in its homogeneity compared to the variability in the LVAV and LVEDV individually, and [15] suggests it may better assess cardiac function in post-MI LVA patients.

Hong and colleagues, in a similarly designed study [16], observed an increase in LV size in end-systolic and end-diastolic phases, with an inverse relationship between the LVAV/LVEDV ratio and EF. A ratio over 16% corresponds to an EF below 50%, with the EF decreasing by 1.1% for each 1% rise in the ratio. The LVAV/LVEDV ratio is a sensitive indicator reflecting heart function in patients with a formed aneurysm.

The aim of this study was to determine LVA characteristics post-myocardial infarction (basal vs. apical) and to evaluate the impact of aneurysm volume in diastole (LVAVd), aneurysm area in diastole (LVAAd), and their respective ratios with the LVEDV and area (LVEDA) on the EF, in order to identify the most critical predictive factors for assessing and managing the negative impact of aneurysms on cardiac function.

## 2. Materials and Methods

This real-world clinical observational study comprised patients with post-infarction LVA who were admitted to the “Dedinje” Cardiovascular Institute in Belgrade, Serbia, for routine transthoracic echocardiographic evaluations. The inclusion criteria were as follows: (i) patients diagnosed with post-infarction LVA undergoing routine transthoracic echocardiography, (ii) patients aged 18 years or older, and (iii) those who could provide informed consent for participation. For a research population of 500 echocardiographic evaluations in post-myocardial infarction patients, where the prevalence of LVA is estimated at 8%, and with a margin of error set at 7% and a confidence level of 95%, the sample size necessary to achieve these parameters was calculated to be 52. All patients underwent percutaneous coronary intervention (PCI) for their myocardial infarction, and the echocardiographic examination was performed one year after myocardial infarction. Ethical approval was secured from the Bioethical Committee of the “Dedinje” Institute to ensure compliance with ethical standards and research protocols. The study adhered to the ethical principles outlined in the Helsinki Declaration, focusing on the protection of patient rights, privacy, and confidentiality throughout the research period.

### 2.1. Data Collection and Patient Classification

Each patient had experienced MI within one year prior to the examination, and all had undergone PCI as part of their treatment. Patients were recruited consecutively as they presented for evaluation or treatment according to the study’s inclusion criteria. This process was designed to minimize selection bias and ensure that the study population accurately reflects the broader patient population encountered in our clinical setting.

Patients underwent transthoracic echocardiography in the left decubitus position according to the standard protocol. LVA was diagnosed through a combination of clinical assessment and imaging techniques. Primarily, two-dimensional echocardiography was employed, which is a standard tool for evaluating myocardial structure and function post-MI. The diagnostic criteria included persistent akinesia or dyskinesia of the myocardial wall in the affected region, as well as the presence of a distinct, thin-walled, dyskinetic segment that bulged outward during systole [17]. While three-dimensional echocardiography could potentially offer more precise measurements of ventricular wall geometry and volume, our study relied on two-dimensional echocardiography due to the unavailability of three-dimensional echocardiography probes in our clinical setting. Two-dimensional echocardiography is a widely established tool in routine clinical practice, providing ample detail for evaluating wall motion abnormalities and post-infarction left ventricular aneurysms. Given the constraints of our available resources, two-dimensional echocardiography was selected as the most practical and suitable method to achieve the study’s objectives. Patients were classified into groups based on LVA characteristics, basal and apical aneurysm location, which were evaluated to understand their impact on cardiac function. Basal aneurysms were defined as those located within the basal segments of the left ventricle, while apical aneurysms were identified in the apical segments. Specifically, 13 patients (24.1%) had basal aneurysms, and 41 patients (75.9%) had apical aneurysms.

The volume of the left ventricular aneurysm (LVAV) was measured in systole (LVAVs) and diastole (LVAVd) from apical views using the area–length method (ml). The aneurysm area (LVAA) was measured in systole (LVAAs) and diastole (LVAAd) from apical views and calculated planimetrically where the boundaries of the aneurysm were traced to obtain the area in cm². End-diastolic and end-systolic volumes of the left ventricle (LVEDV and LVESV) were calculated using the area–length method (ml). End-diastolic and end-systolic areas of the left ventricle (LVEDA and LVESA) were calculated planimetrically (cm²). The left ventricular EF was determined using Simpson’s method (%) [18].

To ensure accuracy and reliability, we assessed both inter- and intra-observer variability for echocardiographic measurements. Two independent observers conducted measurements, showing strong agreement across various parameters, including LVAV volume and aneurysm area. Additionally, repeated measurements by the same observer demonstrated high consistency, confirming the reliable reproducibility of our echocardiographic assessments. These evaluations affirmed the precision and dependability of the echocardiographic data used in this study.

### 2.2. Statistical Analysis

Descriptive statistics were conducted using classical descriptive statistical methods, including the arithmetic mean and standard deviation as a measure of variability. The distribution of numerical variables was tested using the Kolmogorov–Smirnov test to assess normality, and parametric methods were used for variables meeting this criterion. A comparison of baseline characteristics and average values of observed features was performed using the Chi-square test, Student’s T-test, and Mann–Whitney test as a supplementary method in cases of limitations. Correlation analyses were performed using Pearson’s correlation coefficient for parametric data and Spearman’s rank correlation for non-parametric data. A data analysis was performed using the standard statistical program MedCalc (MedCalc Software Ltd., Ostend, Belgium), and a significance level of 0.05 was used in all applied analytical methods.

## 3. Results

The study group consisted of 54 patients with post-infarction LVA, including 36 men (66.7%) with a mean age of 63.19 years and 18 women (33.3%) with a mean age of 65.06 years. The results for the volume and area of the LVA and the volume and area of the LV during systole and diastole are presented with mean, minimum, and maximum values in Table 1.

Table 2 compares values between apical (A) and basal (B) aneurysms regarding aneurysm volume in diastole (LVAVd) and systole (LVAVs), aneurysm area in diastole (LVAAd) and systole (LVAAs), left ventricular volume in diastole (LVEDV) and systole (LVESV), and left ventricular area in diastole (LVEDA) and systole (LVESA). Significantly lower values of aneurysm volume in diastole (LVAVd) (*p* = 0.016), aneurysm area in diastole (LVAAd) (*p* = 0.003), and systole (LVAAs) (*p* = 0.029) were observed in basal aneurysms.

Table 3 presents the values of the ratios of aneurysm volume/left ventricular volume in diastole and systole and the ratios of aneurysm area/left ventricular area in diastole and systole between apical (A) and basal (B) aneurysms. These parameters were significantly lower in basal aneurysms, indicating that basal aneurysms are smaller in size.

The values of EF for apical and basal aneurysms and the values of end-diastolic and end-systolic volumes of the LV are shown in Table 4. There was no significant difference in EF and LV volumes between the compared groups, although the EF and end-diastolic volume of the LV were higher in the basal aneurysm group, while the end-systolic volume was slightly lower.

Table 5 shows the correlation relationships between the EF and the parameters of aneurysm volume and area, LV volume and area, and their respective ratios. There was significant difference between the compared parameters, with the highest correlation between the EF and aneurysm volume in diastole (LVAVd) (*p* < 0.001), slightly lower between the EF and the aneurysm volume to end-diastolic volume ratio (LVAVd/LVEDV) (*p* < 0.001), and the lowest between the EF and LVEDV (*p* < 0.001). Similar correlations were observed between the EF and the mentioned parameters in systole. When comparing the EF with the aneurysm area in diastole (LVAAd) and systole (LVAAs), left ventricular area in diastole (LVEDA) and systole (LVESA), and their respective ratios in diastole (LVAAd/LVEDA) and systole (LVAAs/LVESA), the highest correlation was between the EF and LVAAd, with a lower correlation between the EF and LVEDA, and the lowest between the EF and the LVAAd/LVEDA ratio. Similar relationships were observed between the EF and the specified parameters determined in systole.

## 4. Discussion

Due to the reduction in functional myocardium involved in left ventricular systolic function, LVA is associated with a decrease in the EF. As previously mentioned, the influence of aneurysm volume on heart function, particularly the EF, has been seldom documented in human studies [15]. In experimental studies with rabbits, the ligation of the left anterior descending artery (LAD) and left circumflex (LCx) artery resulted in confirmed aneurysm formation, with the average aneurysm size measuring 33.4% of the left ventricular area as assessed by echocardiography. The decrease in the EF correlated significantly with increased LV dimensions from the apex to the mitral annulus, accompanied by elevated end-diastolic and end-systolic volumes following apical or lateral myocardial infarction, indicating compromised systolic function [19]. Hemodynamic parameters in this study showed a decrease in end-systolic pressure and an increase in end-diastolic pressure of the LV [19].

In another experimental study, Hong and colleagues [15] ligated the LAD and LCx. Four weeks post-procedure, animals were followed using 3D echocardiography to assess the EF and the end-diastolic and end-systolic volumes of the LV and aneurysm volume. They did not find a high correlation between the LVEDV and EF, possibly due to changes in LV geometry due to aneurysm formation, reducing the impact of the LVEDV on heart function. LVAV had a higher correlation with the EF, consistent with the mechanical theory that aneurysm volume reflects myocardial dysfunction. They found that the LVAV/LVEDV ratio had the highest correlation with the EF (r-0.911). This suggests that LVAV/LVEDV reflects cardiac output, with a higher LVAV/LVEDV ratio corresponding to lower cardiac output since the aneurysm cannot empty during systole. The LV ejection is less efficient, leading to a decrease in the EF. Hong and colleagues [15] suggest that the LVAV/LVEDV ratio might be more sensitive than individually observed LVAV and LVEDV in assessing heart function.

In another similarly designed study, Hong and colleagues [16] showed that the LV significantly (*p* < 0.05) increased in end-systolic and end-diastolic phases compared to the control group of rabbits. They demonstrated that the LVAV/LVEDV was inversely correlated with the LV EF. Specifically, an LVAV/LVEDV ratio greater than 16% corresponded to an LV EF of less than 50%. Additionally, a 1.1% decrease in the EF was associated with a 1% increase in the LVAV/LVEDV ratio.

While this study acknowledges that animal models demonstrated a higher correlation between the LVAV/LVEDV ratio and EF, our clinical data revealed a lower correlation. This discrepancy warrants a deeper exploration into the physiological and anatomical differences between animal models and humans that may influence these findings. Animal models, particularly those used in cardiac research, often exhibit more controlled and homogenous conditions compared to human patients. The cardiovascular anatomy and pathophysiology in animals can differ significantly from those in humans. For example, differences in heart size, chamber geometry, and myocardial structure between species can impact the relationship between the LVAV and LVEDV. In animal models, aneurysms may develop in a more uniform manner and be influenced by different hemodynamic and remodeling processes than those observed in humans.

In our study, we compared the correlation between the EF and LVAVd, LVEDV, and the LVAVd/LVEDV ratio. The correlation showed that LVAVd had the highest impact on the EF (−0.695), followed by the LVAVd/LVEDV ratio (−0.637), and the lowest correlation was between the EF and LVEDV (−0.577). Similar relationships were observed when comparing the EF with left LVESV, LVAVs, and the LVAVs/LVESV ratio. When comparing the correlation of the EF with LVEDA, LVAAd, and the LVAAd/LVEDA ratio, the highest correlation was between the EF and LVAAd (−0.718), and the lowest was between the EF and LVAAd/LVEDA (−0.234). Similar correlations were observed between the EF and these parameters in systole.

Our results differ from those of the described experimental studies, which found the highest correlation between the EF and the LVAV/LVEDV ratio (only apical aneurysms were experimentally induced). In our study, 41 patients (75.92%) had apical aneurysms, and 13 patients (24.08%) had basal aneurysms, consistent with the aforementioned studies. The strong negative correlation between the LVAVd and EF underscores that increased left ventricular aneurysm volume during diastole is associated with a diminished EF. This finding suggests that monitoring LVAVd is critical for identifying patients at heightened risk of severe cardiac dysfunction, thereby facilitating early intervention and more aggressive therapeutic strategies. Similarly, the robust negative correlation between the LVAAd and EF demonstrates that a larger aneurysm area correlates with greater impairment in the EF. Prioritizing accurate measurement of LVAAd can enhance clinical decision making regarding surgical intervention or advanced heart failure management, ultimately aiming to mitigate the progression of ventricular dysfunction and optimize patient outcomes.

Comparing the size parameters of apical and basal aneurysms shows significant differences. The LVAVd/LVEDV ratio is significantly higher in apical aneurysms (0.29 vs. 0.11 in basal aneurysms), and similar in systole (0.36 in apical vs. 0.16 in basal aneurysms). The LVAAd/LVEDA is also higher in apical aneurysms (0.39 in diastole vs. 0.29 in basal and 0.43 in systole vs. 0.27 in basal). These differences arise due to significantly larger aneurysm volumes and areas in apical compared to basal aneurysms, while the overall left ventricular volume and area are similar. This means that basal aneurysms are smaller in size. Basal aneurysms typically develop in areas where myocardial damage is less extensive. The basal region of the left ventricle might experience a more localized or less severe infarction, leading to smaller aneurysms. The extent of myocardial necrosis and fibrotic changes in the basal region is generally less compared to the apical region, resulting in smaller aneurysm volumes. The LVAV, as the most variable parameter, is larger in apical aneurysms (84.75 mL) and smaller in basal aneurysms (40.38 mL), making it a critical parameter for assessing heart function according to the mechanical theory. The LVAVd in the overall patient population was 74.07 mL, and the total LVEDV was 270.85 mL.

Our study results show that aneurysm volume has the highest correlation with the LV EF among the tested predictive parameters. In the experimental studies, apical LVA formation was controlled, leading to the conclusion that the LVAV/LVEDV ratio had the highest correlation with the EF. In our clinical study, we included patients with both apical and basal LVA, which is a key reason for the difference in correlation.

This study has several limitations. Firstly, the relatively small sample size may limit our ability to detect significant differences in ventricular aneurysm characteristics based on location. This small sample size could impact the generalizability of our findings and may not fully represent the variability seen in the broader patient population. Secondly, the study’s single-center design may affect the representativeness of our findings. A multi-center study would provide a more comprehensive understanding by capturing a wider range of clinical practices and patient demographics, enhancing the generalizability of the results. Finally, the specific clinical setting and patient population of our study may limit the generalizability of the results to other populations or healthcare settings.

## 5. Conclusions

Basal aneurysms are significantly smaller than apical ones, yet the EF and LV volumes remain similar between the groups, with the EF being slightly higher in the basal group. The predictive parameter of LVAV has the highest correlation with the LV EF. The LVAV/LVEDV ratio shows a lower correlation, and the lowest correlation is between the EF and LVEDV in the presence of a LVA. The EF is reduced in both apical and basal aneurysms but more so in apical aneurysms due to the significantly larger aneurysm volume. The LVEDV is higher in both apical and basal aneurysms (slightly higher in basal aneurysms), while the LVESV is similarly increased in both aneurysm types.

## Figures and Tables

**Table 1:** Results of left ventricular aneurysm volume and area in diastole and systole, and left ventricular volume and area in diastole and systole.

	LVAVd (mL)	LVAVs (mL)	LVAAd (cm²)	LVAAs (cm²)	LVEDV (mL)	LVESV (mL)	LVEDA (cm²)	LVESA (cm²)
mean ± SD	74.07 ± 58.57	67.52 ± 59.97	20.54 ± 10.29	18.93 ± 10.30	270.85 ± 106.85	215.56 ± 170.19	55.09 ± 14.71	45.73 ± 14.24
min	6.00	4.00	4.50	4.30	105.00	65	25.80	25.90
max	260.00	255.00	49.20	45.20	573.00	487	103.40	85.90

LVAVd: left ventricular aneurysm volume in diastole; LVAVs: left ventricular aneurysm volume in systole; LVAAd: left ventricular aneurysm area in diastole; LVAAs: left ventricular aneurysm area in systole; LVEDV: left ventricular end-diastolic volume; LVESV: left ventricular end-systolic volume; LVEDA: left ventricular end-diastolic area; LVESA: left ventricular end-systolic area.

**Table 2:** Results of aneurysm volume and area and left ventricular volume and area during the cardiac cycle in the presence of apical and basal aneurysms.

N = 54	A (41)	B (13)	*p*-Value
LVAVd	84.75 ± 58.82	40.38 ± 44.76	0.016
LVAVs	74.48 ± 58.45	45.57 ± 61.63	0.131
LVAAd	22.70 ± 9.80	13.36 ± 8.73	0.003
LVAAs	20.64 ± 10.12	13.53 ± 9.28	0.029
LVEDV	264.61 ± 96.87	290.54 ± 136.38	0.451
LVESV	216.59 ± 183.16	212.31 ± 183.16	0.938
LVEDA	54.64 ± 13.58	56.49 ± 18.37	0.698
LVESA	45.64 ± 13.04	46.03 ± 18.13	0.931

A: apical aneurysm; B: basal aneurysm; LVAVd: left ventricular aneurysm volume in diastole; LVAVs: left ventricular aneurysm volume in systole; LVAAd: left ventricular aneurysm area in diastole; LVAAs: left ventricular aneurysm area in systole; LVEDV: left ventricular end-diastolic volume; LVESV: left ventricular end-systolic volume; LVEDA: left ventricular end-diastolic area; LVESA: left ventricular end-systolic area.

**Table 3:** The results of the ratios of aneurysm volume to left ventricular volume in diastole and systole, and the ratios of aneurysm area to left ventricular area in diastole and systole, in the presence of apical and basal aneurysms.

N = 54	A (41)	B (13)	*p*-Value
LVAVd/LVEDV	0.29 ± 0.12	0.11 ± 0.08	<0.001
LVAVs/LVESV	0.36 ± 0.18	0.16 ± 0.13	<0.001
LVAAd/LVEDA	0.39 ± 0.10	0.29 ± 0.26	0.044
LVAAs/LVESA	0.43 ± 0.13	0.27 ± 0.08	<0.001

A—apical aneurysm; B—basal aneurysm; LVAVd/LVEDV—ratio of aneurysm volume/left ventricular volume in diastole; LVAVs/LVESV—ratio of aneurysm volume/left ventricular volume in systole; LVAAd/LVEDA—ratio of aneurysm area/left ventricular area in diastole; LVAAs/LVESA—ratio of aneurysm area/left ventricular area in systole.

**Table 4:** The values of ejection fraction, end-diastolic volume, and end-systolic volume of the left ventricle in apical and basal aneurysms.

N = 54	A (41)	B (13)	*p*-Value
EF (%)	23.95 ± 11.16	30.85 ± 14.51	0.770
LVEDV	264.61 ± 96.87	290.54 ± 136.38	0.451
LVESV	216.59 ± 183.16	212.31 ± 126.81	0.938

A—apical aneurysm: B—basal aneurysm; EF—ejection fraction; LVEDV—left ventricular end-diastolic volume; LVESV—left ventricular end-systolic volume.

**Table 5:** Correlation relationships between ejection fraction and aneurysm volume in diastole and systole; aneurysm area in diastole and systole; left ventricular volume in diastole and systole; left ventricular area in diastole and systole; and the ratios of aneurysm volume to left ventricular volume in diastole and systole; and the ratios of aneurysm area to left ventricular area in diastole and systole.

EF		LVEDV	LVAVd	LVAVd/LVEDV
	*p*-value	<0.001	<0.001	<0.001
	Pearson correl.	−0.577	−0.695	−0.637
		LVESV	LVAVs	LVAVs/LVESV
	*p*-value	0.016	<0.001	<0.001
	Pearson correl.	−0.327	−0.678	−0.529
		LVEDA	LVAAd	LVAAd/LVEDA
	*p*-value	<0.001	<0.001	0.088
	Pearson correl.	−0.522	−0.718	−0.234
		LVESA	LVAAs	LVAAs/LVESA
	*p*-value	<0.001	<0.001	0.097
	Pearson correl.	−0.302	−0.682	−0.189

EF: ejection fraction; LVAVd: Left ventricular aneurysm volume in diastole; LVAVs: Left ventricular aneurysm volume in systole; LVAAd: Left ventricular aneurysm area in diastole; LVAAs: Left ventricular aneurysm area in systole; LVEDV: Left ventricular end-diastolic volume; LVESV: Left ventricular end-systolic volume; LVEDA: Left ventricular end-diastolic area; LVESA: Left ventricular end-systolic area LVAVd/LVEDV: ratio of aneurysm volume/left ventricular volume in diastole; LVAVs/LVESV: ratio of aneurysm volume/left ventricular volume in systole; LVAAd/LVEDA: ratio of aneurysm area/left ventricular area in diastole; LVAAs/LVESA: ratio of aneurysm area/left ventricular area in systole.

## Data Availability

Data are available upon request.
